# Capillary Blood GSH Level Monitoring, Using an Electrochemical Method Adapted for Micro Volumes

**DOI:** 10.3390/molecules23102504

**Published:** 2018-09-29

**Authors:** Zaneta Buchtova, Zuzana Lackova, Jiri Kudr, Zdenek Zitka, Jan Skoda, Ondrej Zitka

**Affiliations:** 1 Department of Chemistry and Biochemistry, Mendel University in Brno, Zemedelska 1, CZ-613 00 Brno, Czech Republic; ZanetaBurianova@email.cz (Z.B.); lackova14@seznam.cz (Z.L.); george.kudr@centrum.cz (J.K.); 2 Central European Institute of Technology, Brno University of Technology, Purkyňova 656/123, CZ-612 00 Brno, Czech Republic; 3 University Sports Centre, Faculty of Sports Studies, Masaryk University, Komenskeho namesti 2, CZ-662 43 Brno, Czech Republic; zitka@fsps.muni.cz (Z.Z.); skoda@fsps.muni.cz (J.S.)

**Keywords:** antioxidant molecules, blood drop analysis, electrochemical analysis, nutritional study, sample pretreatment

## Abstract

Glutathione (γ-glutamyl-cysteinyl-glycine; also known as GSH) is an endogenous antioxidant that plays a crucial role in cell defense mechanisms against oxidative stress. It is thus not surprising that this molecule can serve as a biomarker for oxidative stress monitoring. As capillary blood is a highly accessible target for biomarking, it is a valuable bodily fluid for diagnosing human GSH levels. This study focused on the optimization of GSH measurements from micro volumes of capillary blood prior to using electrochemical detection. The optimization of experimental parameters, including the sample volume and its stability, was performed and evaluated. Moreover, we tested the optimized method as part of a short-term study. The study consisted of examining 10 subjects within 96 h of their consumption of high amounts of antioxidants, attained from a daily dose of 2 g/150 mL of green tea. The subjects’ capillary blood (5 μL) was taken at 0 h, 48 h, and 96 h for subsequent analysis. The short-term supplementation of diet with green tea showed an increase of GSH pool by approximately 38% (between 0 and 48 h) within all subjects.

## 1. Introduction

Antioxidants are important molecules that prevent negative effects of free radicals, produced in vivo by molecular oxygen metabolism. The presence of antioxidants and free radicals is well-balanced in healthy organisms, with such compounds being obtained through dietary intake, such as polyphenolics [1], contained in fruit and vegetables [2,3]. Increasing the level of free radicals impairs equilibrium and induces oxidative stress. Oxidative stress can result in the damage of DNA, proteins, lipids, and carbohydrates, and can cause several diseases [4,5,6,7]. Glutathione (L-γ-glutamyl-L-cysteinyl-glycine; also known as GSH) is one of the most important intracellular, non-enzymatic antioxidants [8,9], and is valuable as a pool-making, cofactor substance for the neutralization of reactive oxygen/nitrogen species (ROS, NOS). It plays an important role in nutrient metabolism, cell regulation (including gene expression), DNA and protein synthesis, signal transduction, proliferation and apoptosis, production of cytokines, and immune response [5,10,11,12,13,14]. GSH is also needed for the detoxification of xenobiotics [8,15,16]. GSH possesses thiol moiety, which acts as a reducing agent, due to its ability to transform into oxidized, dimeric glutathione (GSSG), which can be reduced back to GSH in the glutathione-ascorbate cycle [17,18]. Since thiols play an important role in several biological processes, they are objects of increasing interest [19,20]. Additionally, a stable GSH and GSSG ratio is important for the antioxidant capacity of cells, for numerous reasons [12].

Generally, blood markers reliably depict the oxidative status of organisms [21,22]. Usually, venous blood is used for GSH/GSSG analysis; however, a relatively low amount of blood is sufficient for analysis using modern analytical methods. On the other hand, to maintain the selectivity of the detection method in suppressing the interference of other antioxidants, a separation step is necessary. As such, High Performance Liquid Chromatography (HPLC) is commonly used for GSH separation. HPLC can be combined with Electrochemical Detection (HPLC-ED), which is suitable for the analysis of natural antioxidants, such as phenolics, in comparative studies [2,23], or bodily antioxidants in diagnostic studies [22]. HPLC-ED—also frequently used spectrophotometric analysis—is a competitive alternative to laboratory-led procedures, using fluorescence detection [24] or capillary electrophoresis (CE) with Laser Induced Fluorescence (LIF) [25]. It is also possible to analyze tissue or cell samples, using microchip capillary electrophoresis [26] or high performance capillary electrophoresis (HPCE) [27]. Nevertheless, state-of-the-art laboratory analysis is usually carried out by Mass Spectrometry (MS) analysis, which is highly sensitive and selective, in comparison to other methods [28]. However, though MS methods are used in the study of GSH in biochemical studies, they are useless in terms of point-of-care testing (POCT), due to high purchase and maintenance costs, the high level of expertise needed to operate them, and the size of the mass spectrometer itself.

The main reason why we are dealing with electrochemical detection above the other methods previously mentioned is that electrochemical detection has the biggest potential next to optical detection methods, in terms of miniaturization, and thus, in its further use in POCT. For that matter, several recently published works have shown this potential, in using electrochemistry for simple POC-like applications on a real samples [29] or in studies directly focused on biosensors [30].

In addition, colorimetric determination of GSH in blood volumes as low as 0.5 μL was reported by Giustarini et al. [31]. In such cases, capillary blood has been used instead of venous blood, due to the former’s being easier and faster to sample, as well as causing less discomfort to examined patients. Thus, capillary blood sampling has become increasingly more common in medical practice [32]. Though such sampling provides low amounts of blood (about 20 μL), such amounts are sufficient for currently trending POCT devices, which may be developed in the near future. Nevertheless, there is no record regarding the lack of GSH levels in POCT. This may be due to insufficient research into GSH analysis.

Sample preparation plays a crucial role in the analysis of glutathione in blood and other biological materials, as GSH can spontaneously oxidize to GSSG, which can significantly influence the determination of the GSH/GSSG ratio. Several procedures of samples preparation were suggested for GSH/GSSG determination; however, standardized, reliable procedures are lacking [33,34,35,36]. Wurzinger et al. showed that both venous and capillary blood is suitable for determining some oxidative state parameters [37]. Only a few studies have focused on the analysis of GSH/GSSG in capillary blood [38]; one was aimed at the analysis of dried blood spots [39].

Our study focused on the optimization of critical steps in a sample preparation, before GSH/GSSG ratio analysis. To our best knowledge, we are showing the first GSH/GSSG analysis of capillary blood, using an electrochemical method, in a real study in which the short-term effects of a green tea supplementation on the GSH/GSSG ratio were investigated.

## 2. Results and Discussion

### 2.1. Conditions of HPLC-ED Analysis

We used a HPLC-ED set-up, believing it to be the most robust electrochemical method for GSH analysis. To begin, the flow rate of the mobile phase was optimized in the context of our previous work [40]. A flow rate of 1.1 mL min^−1^ was selected as optimal for both GSH and GSSG, as shown in Figure S1A,B, in the Supplementary Materials. The ideal column temperature for determination of GSH and GSSG ratio was also investigated, as shown in Figure S1C,D, in the Supplementary Materials. The best detector temperature for directly influencing GSH and GSSG ratio response was investigated, as well, as shown Figure S1E,F, Supplementary Materials. In light of these findings, the column and detector temperatures were set for 35 °C and 40 °C, respectively.

Direct, accurate analysis of GSH and GSSG was not applicable in this study, due to the rapid self-oxidation of GSH. This required that the sample be protected through freezing or by using stabilizing agents [41]. For most analytical methods analyzing GSH/GSSG ratio, derivatization could be enabled, for a limited time, by derivatizing agents [41]. In the case of direct analysis of GSH/GSSG ratio, without such agents, only HPLC-ED or more rapid, expansive, and accurate ESI-MS/MS [42] methods could be used. However, in such cases, the sample’s temperature needed to be strictly controlled and preanalytical steps taken, before injection of the sample into the system. This needed to be done within a few minutes. As such, the influence of a widely-used, autosampler temperature on the stability of the real sample was investigated. The autosampler temperature of 4 °C provided the most stable response and a gentle increase of the GSH signal was observed, as shown in Figure S2A, in the Supplementary Materials. The best result for preserving the ratio of GSH/GSSG was recorded, while 4 °C was applied, as shown in Figure S2B, in the Supplementary Materials. The best detection potential was found under +0.9 V, as shown in Figure S2C, Supplementary Materials, which is in strong agreement with our previously-published works [40,43].

Additionally, the best conditions for analysis were found, in using: A flow rate of 1.1 mL min^−1^, a column temperature of 35 °C, a detector temperature of 40 °C, a sampler temperature of 4 °C, and an electrode potential of + 0.9 V. The final calibration parameters for the HPLC-ED method were set as follows for GSH–LOD (3S/N) 2.3 μM (linear dynamic range 5.1–325.4 μM, Regression equation y = 0.1581x + 0.2908, RSD 4.5%) and for GSSG–LOD (3S/N) 21.2 μM (linear dynamic range 21.2–172.0 μM, Regression equation y = 0.2276x − 0.0598, RSD 4.8%), as shown in Figure S3C,D, in the Supplementary Materials.

### 2.2. Sample Preparation

As has been reported in previous studies, GSH easily undergoes spontaneous oxidation to GSSG. Therefore, after the optimization of the GSH/GSSG assay, using HPLC-ED, we focused on a real sample preparation before analysis. Next to the strictly-controlled, low temperature, the effect of Trifluoroacetic acid (TFA) (concentrations 5, 10 and 15% *w*/*w*) was examined, based on our previous experience in different studies, focused on HPLC-ED [22,40]. In order to stabilize GSH levels in real samples, 10% (*w/w*) of TFA (final concentration) showed the best results and was used in subsequent experiments, as shown in Figure S3A,B, in the Supplementary Materials.

**Figure 1:**
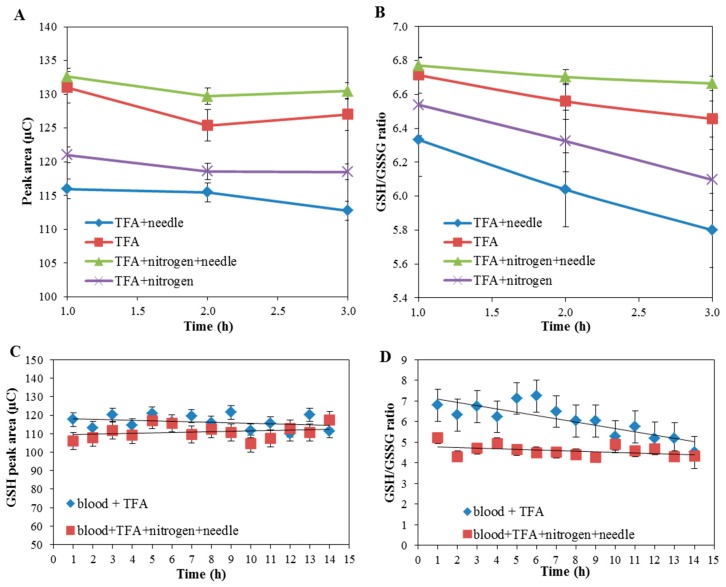
The effect of sample preparation on glutathione (GSH) signal (**A**,**C**) and on the ratio of GSH to oxidized, dimeric glutathione (GSSG) (**B**,**D**) over a certain amount of time. Values are means of three replicates (n = 3). Vertical bars indicate standard error.

Further, we compared the effects of different physical conditions, during sample processing, and their effects on the ratio of GSH/GSSG. TFA (10% *w*/*w*) was added to the sample and divided into 4 aliquots. The first was analyzed without additional processing and others were treated using: (i) liquid nitrogen, (ii) a sonication needle, and (iii) a combination of the two. Subsequently, the samples were centrifuged (at 25,000 RPM, for a 20 min, at 4 °C) and analyzed. The highest levels of GSH were provided by the samples with just the addition of TFA and the addition of TFA, sonication, and nitrogen treatment, as shown in Figure 1A. The most stable GSH/GSSG ratio was provided by the sample treated with TFA, sonication, and liquid nitrogen, as shown in Figure 1B. Subsequently, GSH levels and the GSH/GSSG ratio were observed for a prolonged amount of time (1–14 h), as shown in Figure 1C,D.

From the results obtained, it clearly follows that the best GSH/GSSG ratio was determined using samples prepared with TFA, nitrogen, and sonication needle, and solely TFA, respectively. Moreover, the lower standard error has been reached in the case of samples prepared with TFA, nitrogen, and sonication needle, as shown in Figure 1C,D.

### 2.3. Green Tea Supplementation Study

From the evidence presented, the GSH/GSSG ratio analysis using HPLC-ED methods, coupled with the proper sample pretreatment, could be used in a short-term, preliminary study, on easily acquired capillary blood, during three days of controlled, green tea consumption. In the past, the green tea, *Camellia sinensis*, attracted a lot of attention, due to its positive effects on the antioxidant status of organisms, metabolic syndromes, and cardiovascular diseases [44,45]. Green tea also possesses anti-inflammatory, hepatoprotective, anticancer, and anti-mutagenic properties [46]. Further, epigallocatechin 3-gallate, included in green tea modulates: signals pathways—including NF-κB—influences mitochondrial membrane potential, increases caspase-3 activity, and increases expression of phase II antioxidant enzymes [47,48,49].

**Figure 2:**
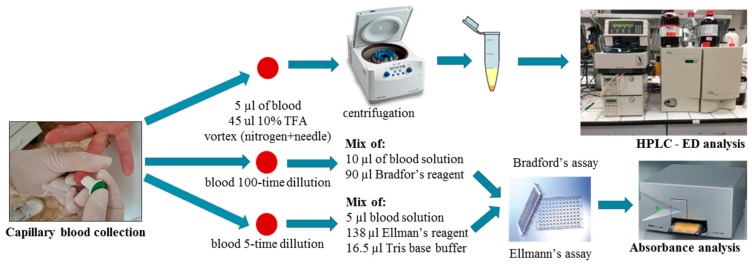
Scheme of glutathione (GSH) and oxidized, dimeric glutathione (GSSH) blood analysis procedure.

Herein, the optimized method for GSH analysis was used to investigate GSH levels in capillary blood samples of group of ten volunteers, supplemented with green tea. This said, the GSSG amount was not the target of the green tea study. GSH levels in volunteers were determined three times: before the start of supplementation, after 48 h of supplementation, and after 96 h of supplementation. The dose of green tea was calculated, according to a volunteer weight. A limited intake of food and beverages with high antioxidant concentrations—like coffee, wine, fruits, vegetables, and dietary supplements—was recommended to volunteers. 15 µL of capillary blood was taken, and GSH levels and total protein concentrations were determined. GSH levels were determined as µg of GSH per total protein mg. The schema of such a sample treatment can be seen in Figure 2.

**Figure 3:**
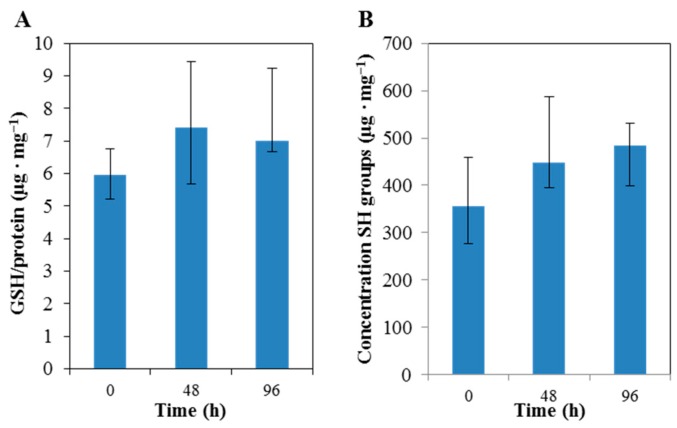
The effect of the tea supplementation on glutathione (GSH) levels (**A**) and the level of total SH-moieties in capillary blood (**B**) (analyzed subjects n = 10). Data are presented as median with error line (the lowest and highest value in the file).

Obtained results suggested that the green tea supplementation was connected to an increase of GSH concentration in blood. Increase of GSH levels by 26% on average was observed between the first and second blood samplings. The third collection of blood showed stagnating, or gently decreasing, GSH levels, as shown in Figure 3. Increasing levels of GSH, after green tea supplementation, has been shown previously in mice, as part of a long-term study [50], and in PC 12 cells under of effect of Pb++ [51]. Basu et al. reported a GSH increase of 34%, after 8 weeks of green tea supplementation [4]. Supplementation with green tea polyphenols in animal models of oxidative stress showed increase in antioxidant enzymes and glutathione concentration [52]. Further, total levels of thiol groups in blood samples were determined, using Ellman’s reagent, as GSH is one of the most represented thiols in blood [53,54,55]. This was determined using 2.5 µL of capillary blood, mixed with phosphate buffer. Similar trends, in terms of GSH levels, were observed total thiol levels. More precisely, after 3 days of the green tea supplementation, the total amount of thiol groups within capillary blood samples increased by an average of 32.6%, but the difference was not so significant, as shown in Figure 3. Moreover, such an intensive increase was not observed. The total amount of thiol groups increased by 33.8% (*p* < 0.05), after 5 days of the supplementation, in comparison with the previous supplementation.

## 3. Materials and Methods

### 3.1. Chemicals

GSH and GSSG, 5,5′-dithiobis-(2-nitrobenzoic acid), cysteine, sodium acetate, Coomasie Brilliant Blue G-250, and a phosphoric acid TFA were obtained from Sigma Aldrich (St. Louis, MA, USA). Methanol in HPLC grade was obtained from Chromservis (Prague, Czech Republic).

### 3.2. Samples

All subjects gave their informed consent for inclusion, before participating in the study. The study was conducted in accordance with the Declaration of Helsinki and the protocol was approved by the Ethics Committee of the Faculty Hospital in Brno (01269746). Blood samples (10–15 μL) were taken from a lateral part of the finger, previously heated in a water bath, using a safety lancet for a capillary blood testing and a Minivette POCT (both from Sarstedt, Nümbrecht, Germany). Directly after, 45 µL of 10% TFA was added to 5 µL of blood and, for a few minutes, the mixture was stored on ice, at 4 °C. Subsequently, samples were placed within liquid nitrogen for 1 min. Samples were melted and disintegrated, using a sonication needle for 30 s, and centrifuged (25,000 RPM, 4 °C). Supernatant (40 μL) was then analyzed, using HPLC-ED.

### 3.3. Method of HPLC-ED

Analysis of GSH and GSSG was performed, using HPLC-ED, which consisted of two chromatographic pumps (Model 582; ESA Inc., Chelmsford, MA, USA), a twelve-channel CoulArray electrochemical detector (Model 5600A; ESA Inc., Chelmford, MA, USA), and a column, containing reverse phase Zorbax eclipse AAA C18 (150 × 4.6 mm; particles size 3.5 µm, Agilent Technologies, Santa Clara, CA, USA). The detector consisted of three, flow analytical chambers (Model 6210; ESA Inc., Chelmsford, MA, USA). Each chamber contained four analytical cells. Each analytical cell contained two reference electrodes (hydrogen-palladium), two counter electrodes, and porous graphite working electrodes. The electrochemical detector, situated in the control module, was tempered. Mobile phase A consisted of TFA-water (3:97, *w*/*w*) and mobile phase B was 100% Met-OH. Compounds were eluted, following a linear increase in gradient: 0 → 1 min (4% B), 1 → 5 min (7% B), 5 → 6 min (98% B), and 6 → 20 min (100% B). Detection was carried out with an applied potential of +0.9 V. The time taken for one analysis was 20 min.

### 3.4. Total Thiol Content Analysis

Blood samples (5 μL, 5-times diluted with water) were mixed with 138 μL of Ellman’s reagent (2 mM 5,5′-dithiobis-(2-nitrobenzoic acid), in 50 mM of sodium acetate). The reaction was started, using an addition of 16.5 μL Tris base buffer (1 M, pH 8 was adjusted, using acetic acid). The colored product of the reaction (159.5 μL) was determined, using Infinite M200Pro (Tecan, Männedorf, Switzerland) at 436 nm, within a 96-well plate with a flat bottom (Thermo Fisher Scientific, Waltham, MA, USA).

### 3.5. Protein Content Analysis

Bovine serum albumin was used as a standard for Bradford’s assay. Bradford’s reagent was prepared as follows: 10 mg of Coomasie Brilliant Blue G-250 was dissolved in 5 mL of 100% ethanol; subsequently, 10 mL of 85% phosphoric acid was added, and the solution was filled to 100 mL with double-distilled water. Ninety microliters of Bradford’s reagent were mixed with blood samples diluted 100 times (10 μL). The colored product of reaction was determined, using Infinite M200Pro (Tecan, Männedorf, Switzerland) at 595 nm. One-hundred microliters were pipetted within a 96-well plate with a flat bottom (Thermo Fisher Scientific, Waltham, MA, USA).

### 3.6. Design of the Study

Ten, healthy volunteers (3 woman and 7 men, between the ages of 23–33 years) were supplemented with green tea. Old England Green tea (Milford, Hall in Tirol, Austria) was prepared as follows: 1 tea bag (2 g of tea mixture) was macerated in 150 mL of water (60 °C) for 4 min. Volunteers were supplemented, according to their weight: 150 mL of tea per 50 kg of weight for one subject, twice a day for one week. Other intakes of antioxidants were not controlled; however, it was recommended that subjects avoid consuming fruits, vegetables, wine, coffee, and dietary supplements. Volunteer’s blood was taken on the first, third, and fifth day of the experiment. Subsequently, the optimized preparation procedure was analyzed, using HPLC-ED. Volunteers signed an informed consent, before the experiment.

## 4. Conclusions

We optimized the direct, electrochemical method for analysis of GSH level in small samples of capillary blood. Suitability of the method was proved by performing a simple experiment, showing the short-term effects of green tea supplementation on GSH levels of 10 volunteers. A fast increase of GSH levels in the capillary blood of volunteers, due to consumption of green tea, was observed. This was in a strong agreement with an observed increase of total thiol concentration in the samples. This is not surprising, as GSH is the most abundant thiol compound in blood. However, we also showed that capillary blood is suitable for GSH electrochemical analysis. Therefore, this method could be used in future, for monitoring changes in GSH levels of capillary blood. In addition, our proposed method will certainly be less demanding on the subjects and patients, in comparison to the intravenous taking of blood by syringe.

## Supplementary Materials

The following are available online at: Figures S1, S2, and S3.
