# Supplementary material for: Capillary Blood GSH Level Monitoring, Using an Electrochemical Method Adapted for Micro Volumes
Source: Molecules. 2018 Sep 29;23(10):2504. doi: 10.3390/molecules23102504 (PMC6222753; doi:10.3390/molecules23102504)
Supplement: Supplementary file 1 [file molecules-23-02504-s001.pdf]

molecules

Article

Capillary Blood GSH Level Monitoring, Using an Electrochemical Method Adapted for Micro Volumes

Zaneta Buchtova[1], Zuzana Lackova[1], Jiri Kudr[1,2], Zdenek Zitka[3], Jan Skoda[3], and Ondrej Zitka [1,2],*

[1] Department of Chemistry and Biochemistry, Mendel University in Brno, Zemedelska 1, CZ-613 00 Brno, Czech Republic
[2] Central European Institute of Technology, Brno University of Technology, Purkyňova 656/123, CZ-612 00 Brno, Czech Republic
[3] University Sports Centre, Faculty of Sports Studies, Masaryk University, Komenskeho namesti 2, CZ-662 43 Brno, Czech Republic
* Correspondence: Ondrej Zitka, Department of Chemistry and Biochemistry, Mendel University in Brno, Zemedelska 1, CZ-613 00 Brno, Czech Republic; E-mail: ondrej.zitka@mendelu.cz; phone: +420-5-4513-3350; fax: +420-5-4521-2044

Assay conditions

In Section 2.1., the optimal setup of the HPLC-ED method, prior to analysis of glutathione (GSH) and oxidized, dimeric glutathione (GSSG), is described.

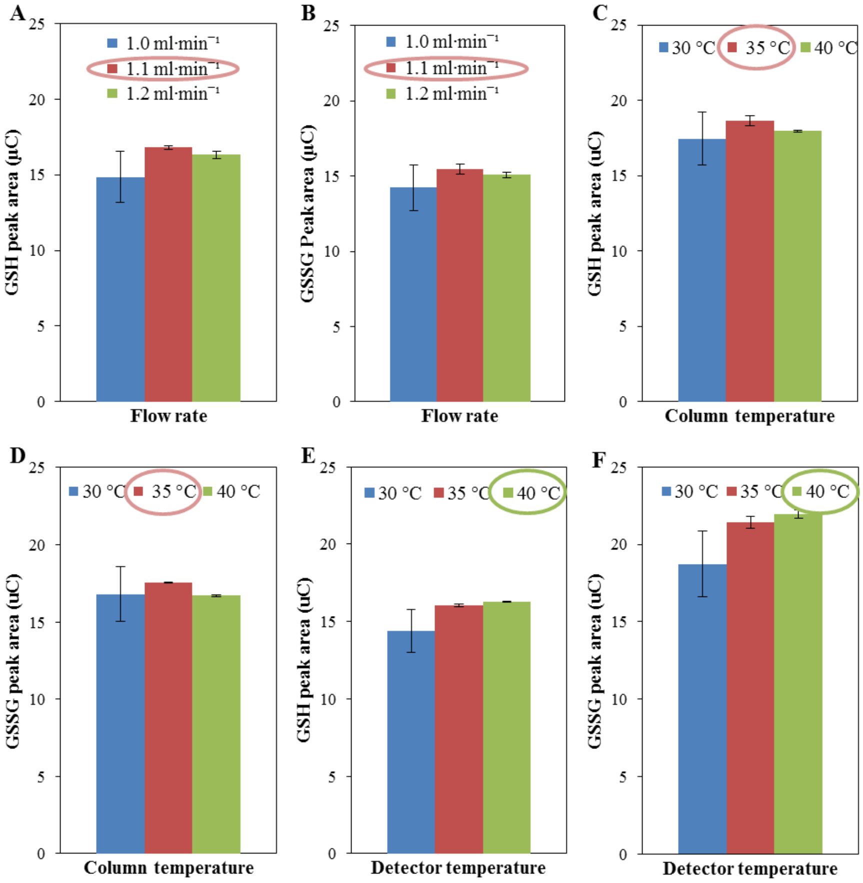

Figure S1. The effect of flow rates (1.0–1.2 ml · min^{-1}) (**A**,**B**), column temperature (30–40 °C) (**C**,**D**), and detector temperature (30–40 °C) (**E**,**F**) on glutathione (GSH) (25 µg·ml^{-1}) and oxidized, dimeric glutathione (GSSG) (80 µg·ml^{-1}) signals, respectively. The values are means of three replicates (n = 3). Vertical bars indicate standard error.

Storage Conditions and Optimal Detection Parameters

The autosampler temperature of 4 and 8 °C provided the best results. A stable response and gentle increase of GSH signal was observed, as shown in Figure S2A, during the period of testing, with a slope of 0.1407x and 0.3561x. A decreasing signal of GSH with a slope of −2.9349x was observed, in the case of samples stored in the autosampler at 12 °C. The GSH and GSSG ratio decreased, during sample storage in the sampler, in all tested cases, as shown in Figure S2,B. Slopes were −0.1122x, −0.1042x, and −0.1359x at 4, 8, and 12 °C, respectively. It is evident that decreases in the case of storage at 4 and 8 °C are comparable, but a decrease higher by nearly 20 % was observed at 12 °C. 4 °C was selected as optimal the sample storage. Several oxidation potentials were used, in order to analyze GSH and GSSG (+0.5 – +1.0 V). As can be seen in Figure S2C, the signal of GSH was almost steadily increasing with the movement of sensing electrodes potentials; however, the GSSG signal rapidly increased at +0.8 and +0.9 V. A potential of +0.9 V was selected as ideal, as it provided the best signal-to-noise ratio for GSSG and acceptable sensitivity for GSH detection.

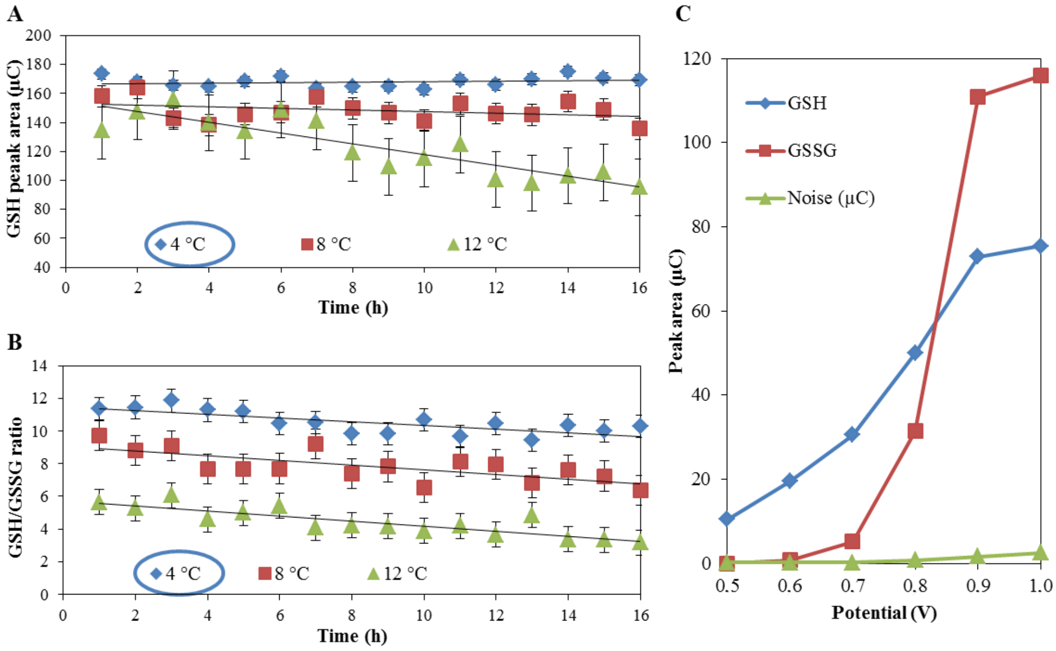

Figure S2. The effect of autosampler (storage) temperature (4–12 °C) on the glutathione (GSH) signal (**A**) and the GSH to oxidized, dimeric glutathione (GSSG) ratio (**B**). The values are means of three replicates (*n* = 3). Vertical bars indicate standard error. The effect of a sensing electrode potential (+0.5 V – +1.0 V) on the GSH and GSSG signals (**C**).

Different concentrations of GSH and GSSG standards in 10% w/w TFA were analyzed, to obtain calibration curves, as shown in Figure S3C. A detailed view of the chromatogram is shown in Figure S3D.

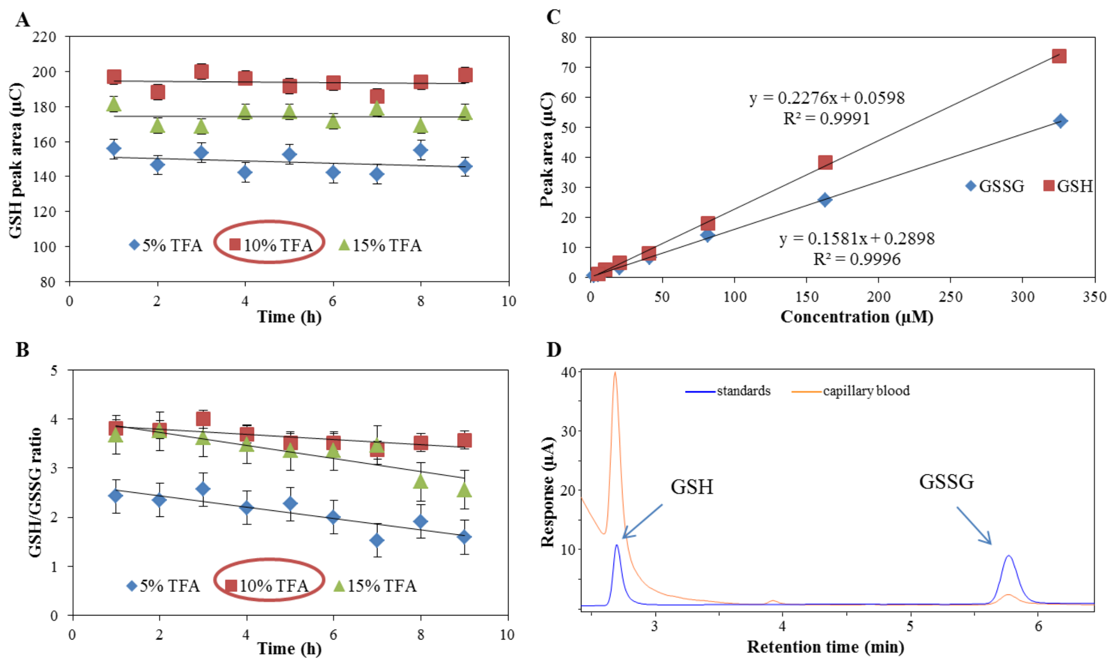

Figure S3. The effect of different TFA concentrations (% w/w) on the signal of glutathione (GSH) (**A**) and the glutathione to oxidized, dimetric glutathione ratio (GSSG) (**B**). The calibration curve of GSH and GSSG was obtained, using the optimized method (**C**). The retention time for GSH was $t_R = 2.5$ min and was $t_R = 5.8$ min for GSSG, in both standard and sample records (**D**).
